# The impact of a native hemiparasite on a major invasive shrub is affected by host size at time of infection

**DOI:** 10.1093/jxb/eraa140

**Published:** 2020-03-18

**Authors:** Robert M Cirocco, José M Facelli, Jennifer R Watling

**Affiliations:** 1 School of Biological Sciences, The University of Adelaide, Australia; 2 Ecology and Environment Research Centre, Manchester Metropolitan University, Manchester, UK; 3 CSIRO Agriculture and Food, Australia

**Keywords:** Alien species, biocontrol, biomass, carbon isotope, chronic photoinhibition, holoparasite, nitrogen, parasitic plants, plant invasions, weed

## Abstract

Many studies have investigated the effect of parasitic plants on their hosts; however, few have examined how parasite impact is affected by host size. In a glasshouse experiment, we investigated the impact of the Australian native hemiparasitic vine, *Cassytha pubescens*, on a major invasive shrub, *Ulex europaeus*, of different sizes. Infected plants had significantly lower total, shoot, and root biomass, but the parasite’s impact was more severe on small than on large hosts. When infected, small but not large hosts had significantly lower nodule biomass. Irrespective of size, infection significantly decreased the host shoot/root ratio, pre-dawn and midday quantum yields, maximum electron transport rates, and carbon isotope composition, and the host nodule biomass per gram of root biomass significantly increased in response to infection. Infection did not affect host foliar nitrogen concentration or midday shoot water potential. Parasite biomass was significantly lower on small relative to large hosts, but was similar when expressed on a per gram of host total biomass basis. Parasite stem nitrogen, phosphorus, and potassium concentrations were significantly greater when *C. pubescens* was growing on small than on large hosts. Our results clearly show that *C. pubescens* strongly decreases performance of this major invasive shrub, especially when hosts are small. This suggests that *C. pubescens* could be used most effectively as a native biocontrol when deployed on smaller hosts.

## Teaser

Native hemiparasitic vine as a novel biocontrol is more effective on smaller invasive shrubs and shows strong promise for protection of biodiversity.

## Introduction

Parasitic plants play important ecological roles in many natural ecosystems (Press and Phoenix, 2005). For instance, they can directly influence nutrient cycling through the production of high-quality litter fall and/or indirectly by promoting the presence of nitrogen (N)-mineralizing bacteria (Bardgett *et al.*, 2006; Quested *et al.*, 2008). More recently, some native parasitic plants are showing promise in helping protect biodiversity by having a greater impact on invasive than on native hosts. For example, in China, the native annual holoparasitic vine *Cuscuta chinensis* has been found to negatively affect performance of invasive but not congeneric native hosts (Li *et al.*, 2012). Also, in Australia, the native hemiparasitic vine *Cassytha pubescens* has been found to strongly affect the health of major invasive leguminous shrubs but not that of the native hosts studied (Prider *et al.*, 2009, 2011; Shen *et al.*, 2010; Cirocco *et al.*, 2018). This differential effect may be underpinned by: (i) parasite haustoria connecting more effectively to the vasculature of invasive hosts and/or (ii) invasive hosts being more effective at acquiring resources than native hosts, with both mechanisms resulting in increased parasite resource supply, growth, and subsequent impact on hosts (Cameron *et al.*, 2006; Cameron and Seel, 2007; Rümer *et al.*, 2006; Li *et al.*, 2012).

Other host traits may also influence the degree of parasite impact, such as host size. Parasitic plants are likely to encounter hosts of different sizes in nature. One might expect that small plants will have lower resource reserves and uptake, and thus supply to parasites, thereby supporting a lower parasite load than larger hosts (Li *et al.*, 2015). The end result may be that a smaller parasite has the same impact on a small host as a larger parasite on a large host (Cirocco *et al.*, 2016*a*). If resource removal is the main mechanism by which the parasite impacts host growth, then following infection it should take longer for a parasite to have a significant effect on a large host than on a small one. However, studies of parasite effects on hosts of different sizes are difficult because controlling for host size can only be achieved in host–parasite systems that lend themselves to glasshouse-type studies. This enables other potentially confounding factors such as host age or dispersal vectors affecting parasite load to be controlled.

Thus, there are very few, if any, studies that have investigated the influence of a parasite on hosts of different sizes. Studies that have used host defoliation (as a proxy for herbivory) offer indirect insights into the response of hosts of different sizes to infection. One study found that irrespective of whether the perennial C_3_ grass *Elymus nutans* was clipped or not, the perennial root hemiparasite *Pedicularis kansuensis* had no effect on host growth, despite the fact that parasite growth was lower on the smaller, clipped hosts (Sui *et al.*, 2015). Similarly, the impact of parasites on host growth was found not to be affected by clipping for the C_4_ perennial grass *Schizachyrium scoparium* and the parasite *Pedicularis canadensis* (Van Hoveln *et al.*, 2011), or for the annual root hemiparasite *Odontites litoralis* ssp. *litoralis* on the perennial grasses *Puccinellia phryganodes* and *Agrostis stolonifera* (Niemelä *et al.*, 2008).

Host size can also be manipulated by changing light supply. In a field study, Borowicz and Armstrong (2012) found that although plant community biomass was lower in shaded plots, the relative negative impact of *P. canadensis* on host biomass was similar in both sun and shade, and light had no effect on parasite growth. Cirocco *et al.* (2016*a*) found that although the host *U. europaeus* was smaller in low-light than high-light treatments, the relative impact of infection with *C. pubescens* was the same in both light conditions. Despite the above examples, to the best of our knowledge there have been no studies that have directly controlled for host size at the commencement of infection.

Here we investigated the impact of a native perennial hemiparasitic vine (*C. pubescens*) on the invasive perennial leguminous shrub (*U. europaeus*), using hosts of different sizes but of the same age. We hypothesized that the impact of the parasite would be more severe on small than on larger hosts. To assess host responses to infection, we measured a number of host traits including growth, photosynthesis, nodulation, water, and nutrient status. We also predicted that growth of *C. pubescens* would be greater on larger hosts, but that parasite load (i.e. parasite biomass per g DW of the host biomass) would be similar regardless of host size.

## Materials and methods

### Study species

*Ulex europaeus* L. (Fabaceae) is an evergreen perennial spiny shrub that can reach 1.5–4 m in height and live for ~20–30 years (Tarayre *et al.*, 2007). It can access N both directly from the soil and via associations with *Bradyrhizobium* species (Rodríguez-Echeverria, 2010). *Ulex europaeus* can produce thousands of seeds per annum that may remain viable in the soil for decades (Hill *et al.*, 2001; Parsons and Cuthbertson, 2001). It is native to Western Europe but has been introduced to all continents and has become a major invasive weed in many parts of the world including Australia (see Hornoy *et al.*, 2013). Indeed, *U. europaeus* is on the world’s 100 worst invasive alien species list (Lowe *et al.*, 2000). *Cassytha pubescens* R. Br. (Lauraceae) is a perennial hemiparasitic vine (~0.5–1.5 mm in diameter) native to Australia that attaches to host stems (Weber, 1981; Kokubugata *et al.*, 2012). It forms numerous ellipsoid haustoria (2–3×1–2.5 mm) that connect to the host xylem, removing water and nutrients (McLuckie, 1924; Weber, 1981). *Cassytha pubescens* seemingly does not show host preference but is typically found infecting perennial species (McLuckie, 1924) including both native and major invasive shrubs such as *U. europaeus*.

### Experimental set-up

In early December 2016, seeds of *U. europaeus* were collected from mature plants located in Engelbrook Reserve (Mt Lofty Ranges of South Australia: 35°01'17''S; 138°45'60''E). In late May 2017, to cue germination, they were immersed in near boiling water and allowed to cool over a 24 h period. Seeds were then sown in 0.22 litre tubes (five seeds per tube thinned to one per tube after germination) containing Mt Compass sand (pH ~4.75). After 6 months, individual seedlings were transplanted into 1.65 litre pots containing the same soil medium. Plants were selected based on height and allocated to two treatments (small or large) which were ~19 cm and 37.5 cm tall, respectively. The height of experimental plants was measured again following the completion of the infection process (see Supplementary Fig. S1 at *JXB* online). There were 20 small and 20 large *U. europaeus* which were randomly assigned to infection treatments (10 infected and 10 uninfected in each height treatment) with the native parasite *C. pubescens*. Plants were infected using the technique of Shen *et al.* (2010). In brief, this involved placing pots with infected *Cytisus scoparius* adjacent to potential hosts. Being a vine with indeterminate growth, the parasite coiled around and attached to the stems of these nearby plants. Once the haustoria were fully developed on the stem(s) of newly infected individuals, the connection from the donor plant was severed. The synchronous infection process was initiated in mid December 2017 and was completed by early March 2018 (~2.75 months duration).

The experiment was conducted in an evaporatively cooled glasshouse at The University of Adelaide (Supplementary Figs S2, S3). Small (S) and large (L) uninfected (–) and infected (+) plants were randomly allocated into 10 blocks, with each block containing one of each treatment combination (e.g. Block 1=S1–, S1+, L1–, and L1+). At this stage, plant height was measured again as mentioned and was significantly different between small and large plants (Supplementary Fig. S1). All experimental plants were well watered and supplied with liquid fertilizer (Nitrosol: Rural Research Ltd, Auckland, New Zealand; NPK 8:3:6) monthly as per the manufacturer’s recommended dosage. Plants within blocks were re-randomized fortnightly to negate any small light differences within the glasshouse. Treatments (infection×size) ran from March 2018 to July 2018 (~4.5 months), after which plants were harvested. Near the end of the experiment (12 d prior to harvesting), the height of plants was measured again (Supplementary Fig. S1).

### Host and parasite photosynthetic performance and water potential (Ψ)

Pre-dawn light use efficiency (*F*_v_/*F*_m_) and rapid light response curves (RLCs) of *U. europaeus* and *C. pubescens* were measured using a MINI-PAM chlorophyll fluorometer (Walz, Effeltrich, Germany) fitted with a leaf-clip (2030-B, Walz). Plants for RLCs were exposed to natural light for ~1.5 h prior to commencing measurements. As RLCs are made up of eight light steps generated by the unit, plants were measured in a shaded area [near darkness: photosynthetic photon flux density (PPFD) ~0–20 μmol m ^– 2^ s ^– 1^] to prevent external light contributions during measurement. RLCs were conducted between 11.00 h and 13.00 h on a sunny day. Light use efficiency (Φ _PSII_) of *U. europaeus* and *C. pubescens* was also recorded at the sixth light step of the RLCs as a proxy for midday Φ _PSII_ (PPFD for host and parasite=984±7 μmol m ^– 2^ s ^– 1^, *n*=48). From the RLCs, the maximum rate of electron transport (ETR_max_) of host and parasite was calculated via regression automatically by the WinControl-3 software (Ver. 3.25; Walz). *F*_v_/*F*_m_ was measured 133 days after treatments had been imposed (DAT), and Φ _PSII_ and ETR_max_ were measured at 132 DAT. *Ulex europaeus* measurements were made on a single spine from each uninfected plant, and single spines from infected shoots on infected plants (*n*=8). Measurements on *C. pubescens* were made 15 cm from the growing tip of the parasite (*n*=8). Blocks 9 and 10 were not included in all measurements (except for comparison between host and parasite water potentials) as these plants appeared suboptimal due to an insect pest.

Midday water potentials (Ψ) of *U. europaeus* and *C. pubescens* were measured with a Scholander-type pressure chamber with digital output (PMS Instrument Company, Albany, OR, USA). Shoots of uninfected plants and infected shoots of infected plants were cut and immediately placed into the chamber, and water potential was recorded when xylem sap first appeared. Parasite stem (15 cm from growing tip) was measured as per above immediately before or after Ψ of its corresponding host was determined. Host water potentials were measured between 140 and 142 DAT (Blocks 1–8; *n*=8). Because of time constraints associated with measuring hosts and subsequent harvesting for biomass, fewer replicates were used for comparing between host and parasite water potentials. Water potentials for parasite:host comparison were made at 142–145 DAT (Blocks 7–10: *n*=4). All Ψ measurements were made between 12.00 h and 14.00 h on sunny or mostly sunny days.

### Host and parasite biomass, δ ^13^C, and nutrient status

Following water potential measurements, a destructive harvest of above-ground *U. europaeus* including *C. pubescens* when present was conducted at 140–142 DAT (*n*=8). Below-ground material (including nodules) was harvested as soon as possible after above-ground biomass at 143–152 DAT. All plant material was oven-dried at 60 °C for 7 d. Carbon isotope composition (δ ^13^C) and N concentration of harvested oven-dried spines from uninfected and infected *U. europaeus* and parasite stems (*n*=8, i.e. Blocks 1–8) were determined with an IsoPrime isotope ratio mass spectrometer (GV Instruments, Manchester, UK) and Isotope CUBE Elemental Analyser (Elementar Analysensysteme, Hanau, Germany) (Flinders Analytical). Inductively coupled plasma spectroscopy (Cuming Smith British Petroleum Soil and Plant Laboratory, Western Australia) was used to measure elemental nutrient concentration of oven-dried host and parasite material.

### Statistical analysis

The variances of the data were homogeneous unless otherwise stated. Full factorial two-way ANOVA was performed on host data. Where no infection×size interaction was detected, independent effects of either infection or size were considered. For example, an independent infection effect compared uninfected plants (small and large uninfected plants pooled) with infected plants (small and large infected plants pooled). Independent size effect compared (small uninfected and infected plants pooled) with large plants (large uninfected and infected plants pooled). One-way ANOVA was used to test the effect of host size on parasite parameters. Degrees of freedom (df), *F*, and sum of square values for host and parasite parameters are presented in Supplementary Tables S1 and S2, respectively. All data were analysed using JMP Ver. 4.0.3 (SAS Institute Inc.) and α=0.05.

## Results

### Host and parasite biomass and photosynthetic performance

**Fig. 1.**
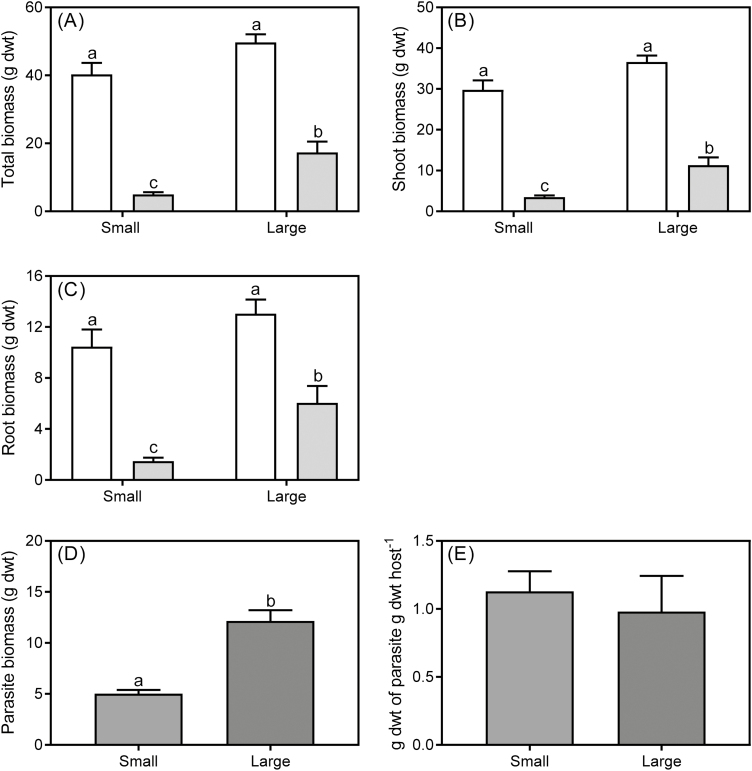
Total (A) shoot (B) and root (C) biomass of small and large *U. europaeus* either uninfected (white bars) or infected (light grey bars) with *C. pubescens*. Total parasite biomass (D) and parasite biomass per unit total host biomass (E) for *C. pubescens* when infecting either small or large *U. europaeus*. Data are means (±1 SE); different letters signify significant differences and *n*=8.

The significant negative effect of infection on host total biomass was more severe for small plants than large ones (infection×size interaction, Table 1). Total biomass of small and large infected plants was 88% and 65% lower, respectively, than that of uninfected plants (Fig. 1A). The infection×size interactions for host shoot and root biomass were marginally significant (Table 1). These marginally significant effects were confirmed by the conservative Tukey’s HSD (honestly significant difference) pairwise comparison test detecting significant differences among treatments for both shoot and root biomass, and should not be ignored (Facelli and Facelli, 2002). Shoot biomass of infected small and large plants was 88% and 69% lower than that of uninfected plants, respectively (Fig. 1B). Infection significantly decreased root biomass of small and large plants by 86% and 54%, respectively (Fig. 1C). Parasite total biomass was significantly affected by size of *U. europaeus* (*P*<0.0001; data log transformed for homoscedasticity). Parasite total biomass on large hosts was ~60% greater than that growing on small hosts (Fig. 1D). However, the size of *U. europaeus* did not significantly affect parasite biomass on a per gram of host total biomass basis (*P*=0.631; Fig. 1E).

**Table 1.** *P*-values for independent effects of infection with *C. pubescens* (I), size of *U. europaeus,* and their interaction (I×S) on total, shoot, and root biomass, shoot/root ratio (S/R), nodule biomass (Nod), Nod g^–1^ host root biomass, pre-dawn and midday quantum yields (*F*_v_/*F*_m_, Φ _PSII_), maximum electron transport rates (ETR_max_), midday water potential (Ψ), carbon isotope composition (δ ^13^C), and foliar nitrogen (N) and iron (Fe) concentration of *U. europaeus*

	Total	Shoot	Root	S/R	Nod	Nod g^–1^ root	*F*_v_/*F*_m_	Φ_PSII_	ETR_max_	Ψ	δ^13^C	N	Fe
I	**<0.0001**	**<0.0001**	**<0.0001**	**0.020**	**<0.0001**	**0.013**	**0.0004**	**0.026**	**0.003**	0.779	**0.0007**	0.925	**0.001**
S	**<0.0001**	**<0.0001**	**0.0005**	0.193	**0.011**	0.105	0.172	**0.041**	0.113	**0.043**	**0.002**	0.228	**0.039**
I×S	**0.053**	**0.066**	**0.068**	0.559	**0.029**	0.662	0.832	0.394	0.415	0.235	0.184	0.742	**0.0005**

Significant and marginally significant effects are in bold; df, *F*, and sum of square values are presented in Supplementary Table S1. Total, shoot, root, and Nod biomass were square root transformed, and ETR_max_, Nod g^–1^ host root biomass, and Fe were log transformed to achieve homoscedasticity.

Regarding other host growth measures, infection significantly decreased the shoot:root ratio by 22% (no interaction: Tables 1, 2). Infection significantly decreased nodule biomass of small plants but not that of large ones (infection×size interaction, Table 1). Nodule biomass of small and large infected plants was 75% and 37% lower, respectively, than that of uninfected plants (Table 2). There was no infection×size interaction found for host nodule biomass when expressed on a per gram of host root biomass basis, but this parameter was independently affected by infection (Table 1). In this case, infection significantly increased nodule biomass per gram of host root biomass by 44% (Table 2).

**Table 2.** Shoot/root ratio (S/R), nodule biomass (Nod; g DW), Nod per gram of host root biomass, midday water potential (Ψ; MPa), and carbon isotope values (δ ^13^C; ‰) of small (S) or large (L) *U. europaeus* either uninfected (minus) or infected (plus) with *C. pubescens*

	S/R	Nod	Nod g^–1^ root	Ψ	δ^13^C
Treatment					
S–	3.09±0.319	0.487±0.057 a	0.049±0.004	−1.44±0.082	−31.6±0.223
S+	2.59±0.301	0.120±0.018 b	0.100±0.020	−1.30±0.094	−32.8±0.240
L–	2.89±0.169	0.511±0.050 a	0.039±0.003	−1.53±0.105	−31.2±0.285
L+	2.07±0.258	0.323±0.061 a	0.058±0.007	−1.62±0.105	−31.7±0.141
Infection					
–	2.99±0.176 a	N/A	0.044±0.003 a	−1.48±0.056	−31.4±0.185 a
+	2.33±0.203 b	N/A	0.079±0.011 b	−1.43±0.067	−32.3±0.196 b
Size					
S	2.84±0.222	N/A	0.074±0.012	−1.37±0.063 a	−32.2±0.220 a
L	2.48±0.183	N/A	0.049±0.004	−1.58±0.073 b	−31.4±0.170 b

Data are means (± 1 SE). Treatments: *n*=8, infection or size: *n*=16, and different lower case letters signify a significant difference. Significant infection×size interaction for nodule biomass, independent infection effect on S/R, Nod g^–1^, root, and δ ^13^C, and significant independent size effect on Ψ and δ ^13^C.

**Fig. 2.**
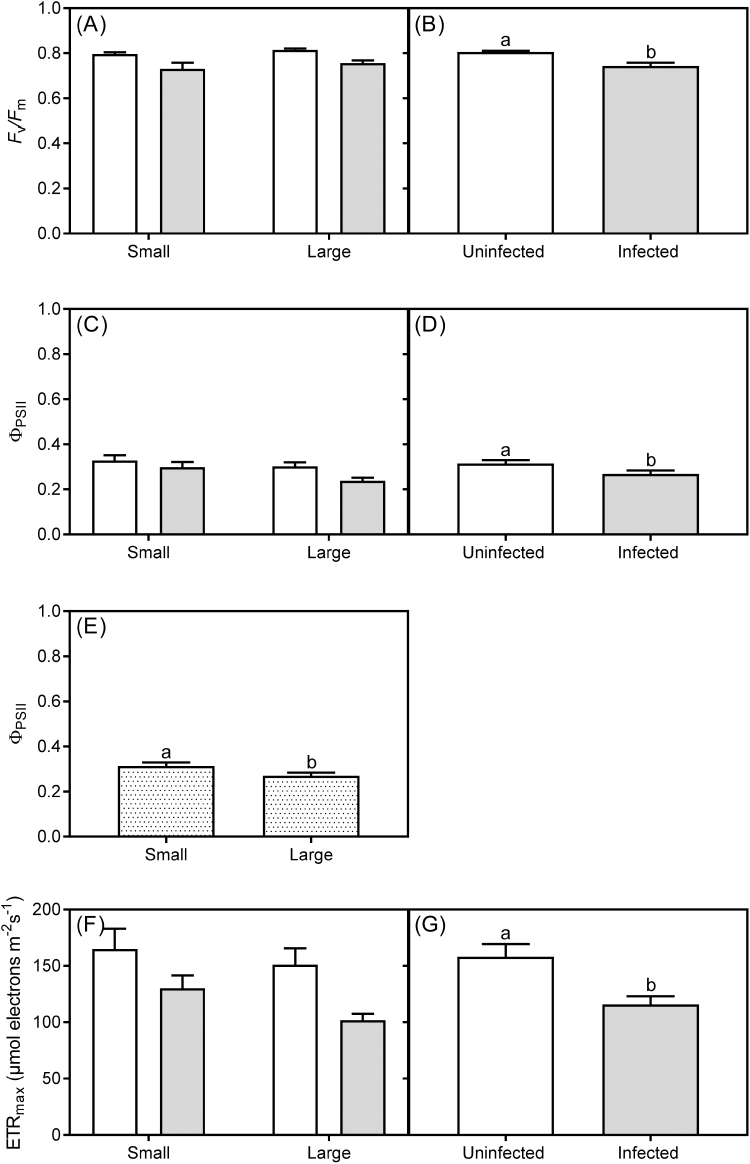
(A) Pre-dawn (*F*_v_/*F*_m_) and (C) midday quantum yield (Φ _PSII_), and (F) maximum electron transport rate (ETR_max_) of small and large *U. europaeus* either uninfected (white bar) or infected (light grey bar) with *C. pubescens*. Independent effect of infection on (B) pre-dawn and (D) midday quantum yield, and (G) maximum electron transport rate of *U. europaeus*. Independent effect of size on (E) midday quantum yield of host (dotted bars). Data are means (±1 SE); different letters signify significant differences. (A, C, F) *n*=8 and (B, D, E, G) *n*=16.

**Fig. 3.**
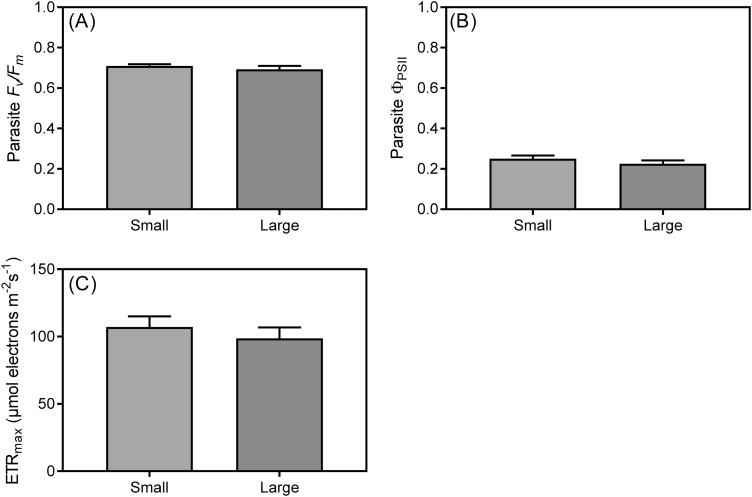
(A) Pre-dawn (*F*_v_/*F*_m_) and (B) midday quantum yield (Φ _PSII_), and (C) maximum electron transport rate (ETR_max_) of *C. pubescens* when infecting small or large *U. europaeus*. Data are means (±1 SE); no significant differences and *n*=8.

There were no interactions between infection status and host size for *F*_v_/*F*_m_, Φ _PSII_, or ETR_max_ of *U. europaeus*, but they were all independently affected by infection (Table 1; Fig. 2A, C, F). Host *F*_v_/*F*_m_, Φ _PSII_, and ETR_max_ were 8, 15, and 27% lower, respectively, than for uninfected plants (Fig. 2B, D, G). There was also an independent effect of size on Φ _PSII_ of *U. europaeus*, with large plants having 13% lower Φ _PSII_ than small plants (Table 1; Fig. 2E). Size of the host had no influence on *F*_v_/*F*_m_ (*P*=0.382), Φ _PSII_ (*P*=0.293), or ETR_max_ (*P*=0.470) of *C. pubescens* (Fig. 3A–C).

### Host and parasite Ψ, δ^13^C, and nutrient status

There was no infection×size interaction detected for Ψ of *U. europaeus*; however, there was an independent effect of host size on this parameter (Tables 1, 2). Water potential of small plants was 13% less negative than that of large plants (Table 2). Water potential (MPa) of *C. pubescens* was not affected by host size (*P*=0.865) and was –1.54±0.089 and –1.57±0.176, on small and large infected hosts, respectively. There was no significant difference between Ψ (MPa) of infected plants –1.43±0.081 and parasite –1.55±0.092, regardless of host size (species effect: *F*_1, 11_=1.40; *P*=0.262, *n*=8).

**Fig. 4.**
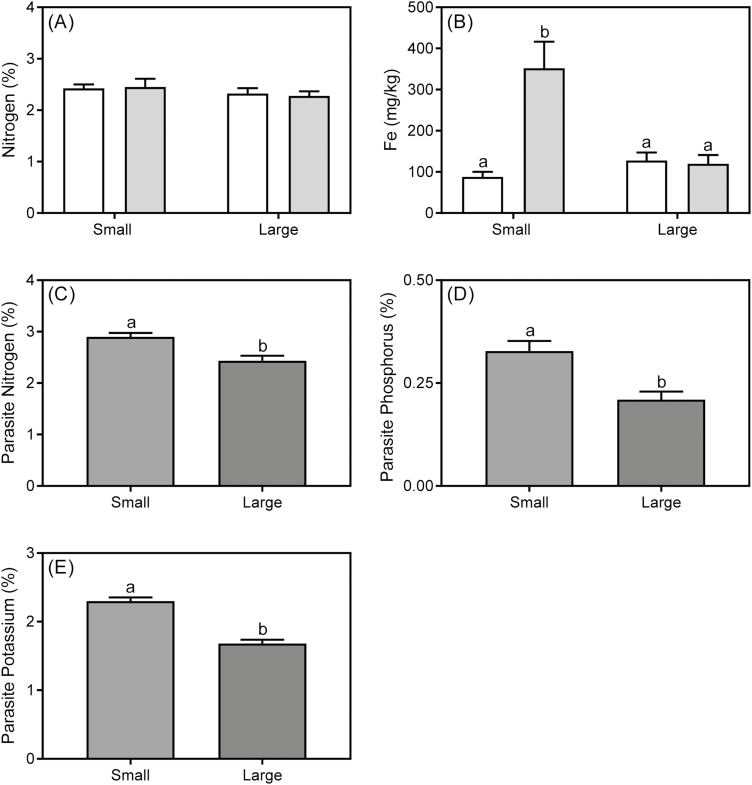
Spine nitrogen (A) and iron (B) concentration of small or large *U. europaeus* when uninfected (white bar) or infected (light grey bar) with *C. pubescens*. (C) Nitrogen, (D) phosphorus, and (E) potassium concentration of stems of *C. pubescens* when infecting small or large *U. europaeus*. Data are means (±1 SE); different letters signify significant differences, (A, C) *n*=8 and (B, D, E) *n*=4.

Regarding δ ^13^C of *U. europaeus*, no infection×size interaction was found but this host parameter was independently affected by both infection and size (Tables 1, 2). δ ^13^C of *U. europaeus* significantly decreased as a result of infection (Table 2). On average, δ ^13^C of small *U. europaeus* was significantly lower than that of large *U. europaeus* (Table 2). δ ^13^C (‰) of *C. pubescens* was not affected by host size (*P*=0.303) and was –28.8±0.245 and –28.5±0.166 on small and large hosts, respectively. However, δ ^13^C was significantly different between host and parasite (*F*_1, 28_=314; *P*<0.0001). δ ^13^C (‰) of infected *U. europaeus* (–32.3±0.196) was significantly lower relative to that of *C. pubescens* (–28.7±0.149), regardless of host size (*n*=16). There were no significant treatment effects found for foliar tissue N concentration of *U. europaeus* (Table 1; Fig. 4A). However, an infection×size interaction was found for host Fe concentration (Table 1). Infection significantly increased Fe of small plants by 75%, whereas the parasite had no effect on Fe concentration of large plants (Fig. 4B). Host size significantly affected the concentration of N (*P*=0.002), phosphorus (*P*=0.010), and potassium (*P*=0.0002) in the parasite. N, phosphorus, and potassium concentrations in parasite stems on small hosts were 16, 36, and 27% higher, respectively, than those supported by large hosts (Fig. 4C–E).

## Discussion

Supporting our hypothesis, plants infected with *C. pubescens* had significantly lower growth and nodulation than uninfected *U. europaeus*, but the effects were greater when hosts were small. Small hosts also supported significantly less parasite biomass than larger hosts, although parasite biomass per gram of DW of the host was similar for both size treatments.

Total and shoot biomass of infected, small *U. europaeus* were both 88% lower than for uninfected plants, whereas the differences for large infected plants were 65% and 69%, respectively. Similarly, Li *et al.* (2015) found that the effect of *Cuscuta australis* on host total biomass was significantly greater for younger *Bidens pilosa* than for older hosts. We also found that host root biomass was significantly lower when infected with *C. pubescens*, but also more severely so for small plants (86%) than large plants (54%). Again, a similar result was reported by Li *et al.* (2015), where root biomass of young (but not older hosts) was significantly lower than for uninfected *B. pilosa*. We found that infection significantly diminished growth of large plants (albeit less severely), whereas Li *et al.* (2015) found that infection had no significant effect on growth measures of the oldest (largest) *B. pilosa*. This discrepancy between findings may be due to the parasite negatively affecting photosynthetic performance of both small and large hosts in our study, whereas in Li *et al.* (2015) the parasite only affected photosynthesis of younger plants. It might also be due to plants in our experiment being infected for nearly four times longer than those in Li *et al.* (2015). Here, the stronger infection effect on small plants may be due to small plants having higher water availability and water potential, making it easier for the parasite to extract resources. Indeed, soil in pots containing small plants retained water for longer (personal observation), small plants were more profligate in their water use (as indicated by significantly lower δ ^13^C: size effect, Table 1), and had significantly higher Ψ than large plants (Table 2). All of the above would have facilitated removal of resources by the parasite. Parasite stems were significantly enriched in nutrients when growing on small rather than on large hosts (Fig. 4C–E). This is supported by earlier work in which *C. pubescens* more severely affected growth of *U. europaeus* in high relative to low water conditions (Cirocco *et al.*, 2016*b*). The stronger effect on small plants may also have been due to smaller plants having lower resource acquisition and initial reserves than large hosts, resulting in greater sensitivity to infection.

Large hosts supported twice as much total parasite biomass as small hosts. Li *et al.* (2015) also found that parasite biomass significantly increased with increasing age and size of *B. pilosa*. It is likely that larger hosts would have a greater capacity for resource supply to the parasite, explaining why parasites in both studies grew more on larger plants (Li *et al.*, 2015). Nutrient and water supply is likely to be a major determinant of growth, particularly in parasitic vines with indeterminate growth such as *Cassytha* and *Cuscuta*. This is further supported by the fact that parasite biomass per gram of DW of the host was similar for both the small and large hosts in our study (Fig. 1E). In contrast, Li *et al.* (2015) found that *Cuscuta australis* biomass per gram of DW of the host was significantly higher on younger than on older (larger sized) hosts. This was probably due to the lack of any significant effect of the parasite on host biomass of older *B. pilosa*. In our study, one might expect parasite biomass per gram of DW of the host to be higher on large plants as their biomass was less affected by infection. The fact that this was not the case may be due to large plants having significantly lower Ψ, thereby making it more difficult for the parasite to extract resources. There is a possibility that the parasite might be able to adjust its resource acquisition depending on host size (Kabiri *et al.*, 2017), or a combination of both host and parasite regulation of resource transfer, explaining our finding.

As with host growth, *C. pubescens* had a greater impact on nodule biomass of small hosts. This contrasts with the results of Cirocco *et al.* (2016*b*) who found that although the parasite more severely affected growth of *U. europaeus* in high relative to low water conditions, host nodule biomass was similarly impacted irrespective of water supply. In another study, we found that growth and nodule biomass of *U. europaeus* were both negatively affected by *C. pubescens* regardless of N supply (Cirocco *et al.*, 2017). Studies have found that parasitic plants affect host nodulation in some cases but not others (e.g. Tennakoon *et al.*, 1997; Gao *et al.*, 2019; Sui *et al.*, 2019). Here, nodule biomass of small hosts may have been lower simply because there was less root biomass as a result of infection.

Nodule biomass (Nod) per gram of host root DW was significantly increased by infection, regardless of host size. In contrast, Cirocco *et al.* (2016*b*) found that *U. europaeus* infected with *C. pubescens* had significantly lower Nod per gram of host root DW than uninfected plants. On the other hand, Cirocco *et al.* (2017) found no difference between Nod per gram of host root DW of infected and uninfected plants. It is unclear why these results differ. Here, although no interaction was found, Nod per gram of host root DW was almost twice as high in small hosts as in large ones (Table 2). The higher Nod per gram of host root DW of the small hosts may have resulted in higher rates of N fixation per gram of root biomass in response to N removal by the parasite (Fig. 4C). This presumably greater engagement with rhizobia could lower soil pH around the host roots, leading to increased mobility of iron (Tang *et al.*, 1999; Houmani *et al.*, 2015). This may explain the 75% higher iron concentration in spines of small hosts relative to large hosts and uninfected plants (Fig. 4B). Similarly, significant increases in iron and aluminium of *U. europaeus* in response to *C. pubescens* have been consistently found across three sites in the field (Cirocco *et al.*, 2018).

The effects of *C. pubescens* on host growth and nodulation may in part be explained by significant infection effects on host photosynthesis (proxy: ETR_max_), irrespective of host size (Fig. 2G). *Cassytha pubescens* has also previously been reported to negatively affect photosynthesis of a number of invasive hosts, including *U. europaeus* (Prider *et al.*, 2009; Shen *et al.*, 2010; Cirocco *et al.*, 2016*a*, 2017, 2018). In contrast, Li *et al.* (2015) found that *Cuscuta australis* significantly affected photosynthesis of young hosts but not that of older ones. Examples from other systems generally show that holoparasites (e.g. *Orobanche* and *Cuscuta*) can increase or decrease host photosynthesis while hemiparasites decrease (e.g. *Striga*) or have no discernible effect on this process (Johnson and Choinski, 1993; Seel and Press, 1996; Watling and Press, 2001; Hwangbo *et al.*, 2003; Reblin *et al.*, 2006). Host photosynthesis decreasing in response to infection is typically attributed to parasite-induced nitrogen and or stomatal limitations (Taylor *et al.*, 1996; Chen *et al.*, 2011; Jokinen and Irving, 2019). In our study, it is not clear why photosynthesis was lower in infected plants as host Ψ and N status were unaffected by infection. Also, infected plants had significantly lower δ ^13^C than uninfected plants (Table 2), suggesting that infection did not trigger a decrease in host stomatal conductance.

Lower rates of host photosynthesis resulting from infection would have led to an increase in the ratio of PPFD to photosynthesis, thereby creating conditions of excess light (Demmig-Adams and Adams, 1992). Prolonged plant exposure to excess light can result in chronic photoinhibition, as indicated by decreases in *F*_v_/*F*_m_ (Demmig-Adams and Adams, 2006). Here, host *F*_v_/*F*_m_ was significantly lower than that of uninfected plants, regardless of host size. *Cassytha pubescens* also significantly decreased *F*_v_/*F*_m_ of *U. europaeus* both in the field, and irrespective of water availability, in the glasshouse (Cirocco et al., 2016*b*, 2018). In glasshouse but not field conditions, *C. pubescens* significantly decreased *F*_v_/*F*_m_ of the invasive host *Cytisus scoparius* (Prider *et al.*, 2009; Shen *et al.*, 2010). However, this native parasite has not been found to affect *F*_v_/*F*_m_ of any native hosts studied so far (Prider *et al.*, 2009; Cirocco *et al.*, 2015). Significant declines in *F*_v_/*F*_m_ can translate into strong decreases in host C over time (Gurney *et al.*, 2002) and thus, along with effects on maximum rates of photosynthesis, may also explain why infection decreased growth and nodulation of both small and large hosts.

As mentioned, infected *U. europaeus* had significantly lower δ ^13^C than uninfected plants, regardless of host size. Similar results have been reported for this host in both field and glasshouse experiments (Cirocco *et al.*, 2016*b*, 2018). However, the difference between δ ^13^C of infected and uninfected *U. europaeus* in the current study was twice as large (1.2‰) for small plants than for large ones (0.5‰). These findings suggest that *U. europaeus*, particularly when small, is more profligate in its water use. This response may be triggered by higher soil water availability due to the smaller size of plants, and may compensate to some degree for resource removal by the parasite. δ ^13^C of *C. pubescens* was significantly higher than that of the host, irrespective of size, as similarly found by Cirocco *et al*. (2016*b*, 2018). In contrast, Scalon and Wright (2015) found that mistletoes typically maintain lower δ ^13^C than their hosts, particularly in warmer environments. The higher δ ^13^C of *C. pubescens* suggests that the parasite is more conservative in its water use than its host which may be a consequence of being leafless and having much lower stomatal density than hosts. It might also signal a degree of parasite heterotrophy (Cernusak *et al.*, 2004).

### Conclusion

In line with our hypothesis, *C. pubescens* had a greater impact on total, shoot, root, and nodule biomass of small plants relative to large ones. The stronger effects of infection on small hosts could be explained by small plants having higher water availability thereby enabling greater removal of resources by the parasite. Although parasite stems on small hosts were nutrient enriched relative to those on large hosts, parasite biomass per gram of DW of the host of small plants was no different from that of large plants. Parasite growth on small hosts was possibly constrained by the effects of infection on host roots and nodules (probably restricting resource acquisition) despite them having almost double the nodules per gram of roots of large infected hosts. Thus, as predicted, parasite growth seems tightly regulated by host growth. In addition, effects on physiological processes (e.g. photosynthesis) may in part also help explain why hosts of both sizes were affected by infection. Future studies should include investigating the effect of this native parasite on hosts of different sizes in a natural setting. For example, a thicket of *U. europaeus* very large in size may support very large parasite biomass (Supplementary Fig. S4) and the associated impact of *C. pubescens* may be similar to that of smaller parasites on smaller plants. However, it may take longer for the parasite to exert a negative effect on large plants which in part may also explain why they were more tolerant to infection in our study. Our data continue to support the potential use of this novel native biocontrol and that it could be particularly effective when invasive shrubs are smaller in size. For applied purposes this may entail targeting parasite deployment on invasive shrubs either soon after germination or following mechanical pruning. Plant invasions are one of the major threats to global biodiversity (Vilà *et al.*, 2011). If successful, *C. pubescens* could be used to help mitigate the devastating economic and environmental impacts of invasive shrubs and play a key role in biodiversity protection.

## Supplementary data

Supplementary data are available at *JXB* online.

Fig. S1. Mean heights of small and large uninfected and infected plants at the start and end of the experiment, and associated *P*-values.

Fig. S2. Photos of the experiment and large uninfected and parasite-infected plants.

Fig. S3. Photos of small uninfected and parasite-infected plants.

Fig. S4. *Cassytha pubescens* ‘infection front’ moving over a large thicket of *Ulex europaeus* in the Mt Lofty Ranges of South Australia.

Table S1. *F* and sum of square values for parasite infection and host size effects on host parameters.

Table S2. *F* and sum of square values for host size effects on parasite parameters.

## Supplementary Material

eraa140_suppl_Supplementary_Material
