# Supplementary material for: The impact of a native hemiparasite on a major invasive shrub is affected by host size at time of infection
Source: J Exp Bot. 2020 Mar 18;71(12):3725–34. doi: 10.1093/jxb/eraa140 (PMC7307848; doi:10.1093/jxb/eraa140)
Supplement: eraa140_suppl_Supplementary_Material [file eraa140_suppl_supplementary_material.pdf]

Journal of Experimental Botany

The impact of a native hemiparasite on a major invasive shrub is affected by host size at time of infection

Robert M. Cirocco, José M. Facelli, Jennifer R. Watling

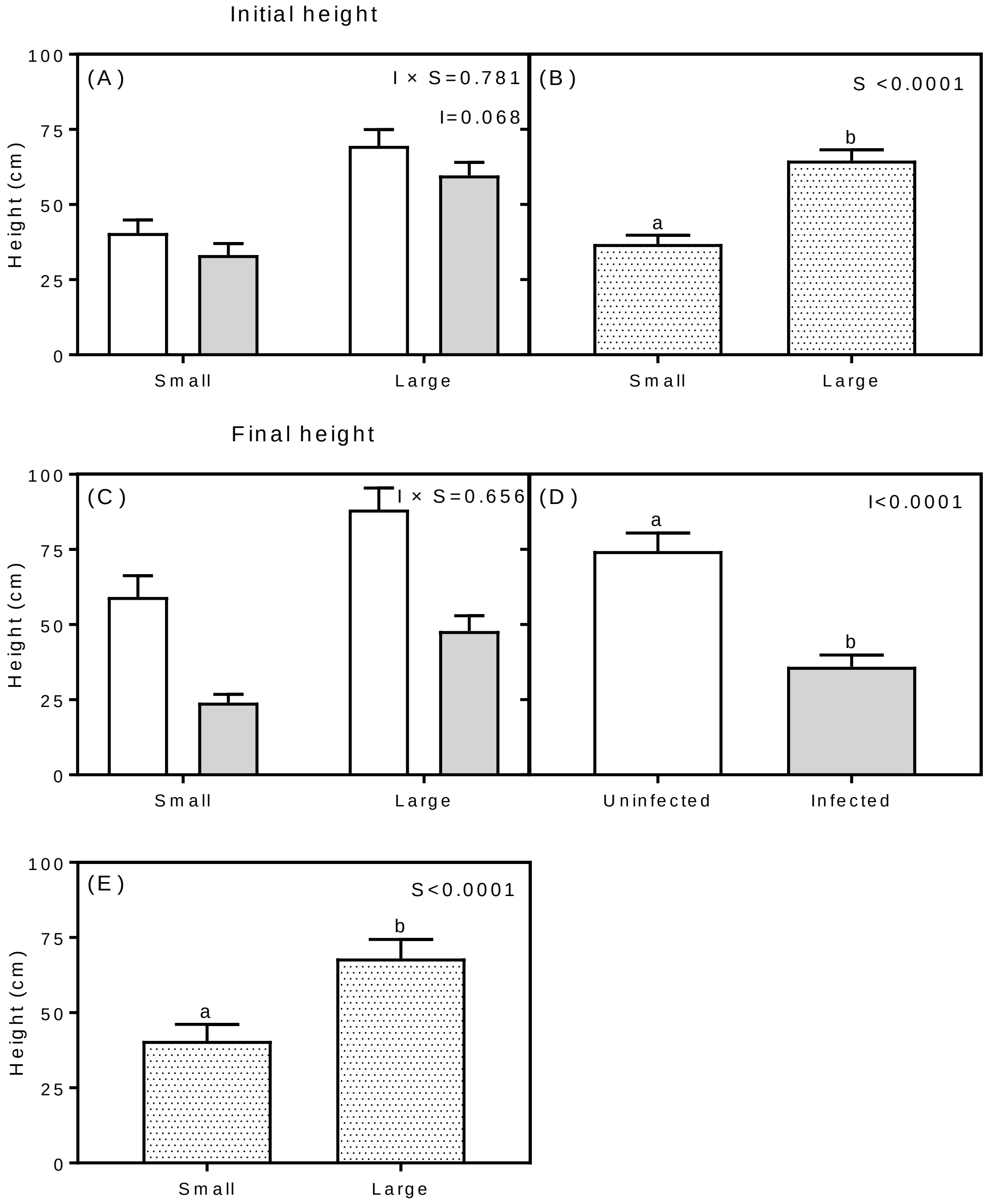

Figure S1. Height of small and large *U. europapeus* either uninfected (white bar) or infected with *C. pubescens* (light grey bar) at the beginning (A) and at end of the experiment (C).

Independent effect of size (B) on height of *U. europaeus* at start of experiment (dotted bars). Independent effect of infection (D) and size (E) on host height at end of experiment. Data are means (±1 SE), (A, C) n=9–10 and (B, D, E) n=19–20, different letters signify significant difference and P-values are displayed in panels for infection (I) × size (S) interaction and independent I and S effects.

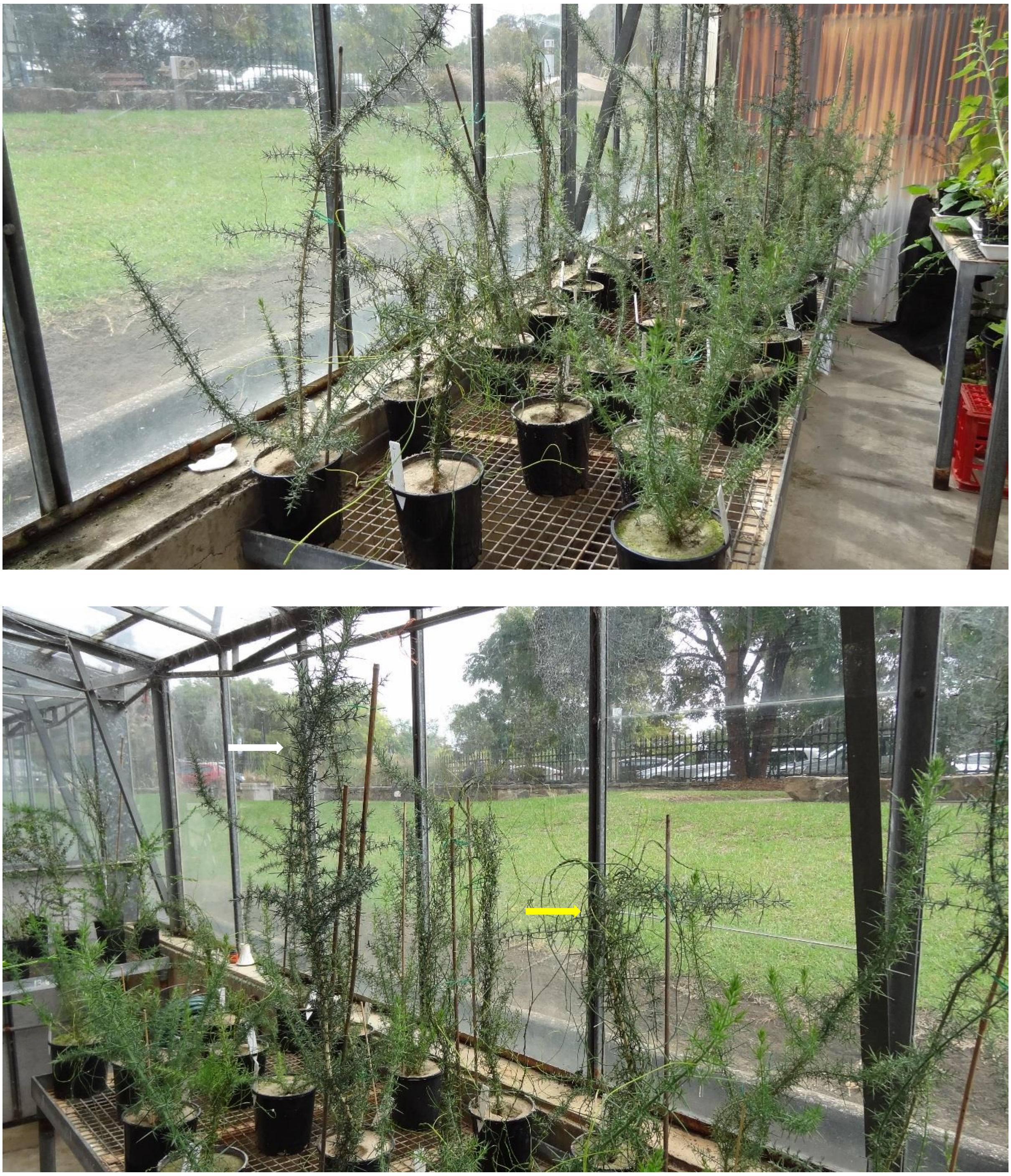

Figure S2. Top: photo of the experiment; Bottom: large uninfected *Ulex europaeus* (white arrow) and *U. europaeus* infected with *Cassytha pubescens* (yellow arrow).

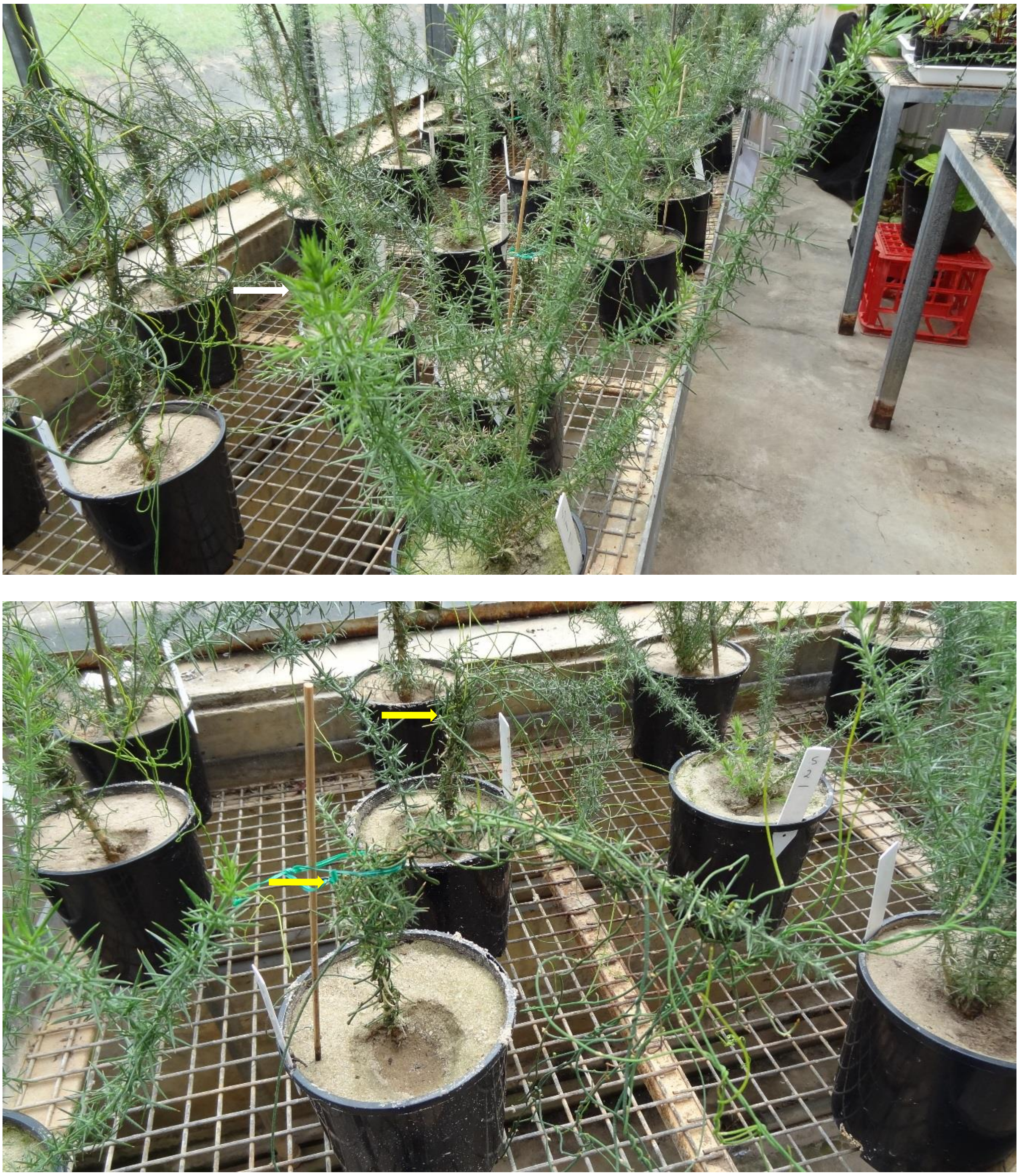

Figure S3. Top: small uninfected *U. europaeus* (white arrow); Bottom: small infected *U. europaeus* (yellow arrows).

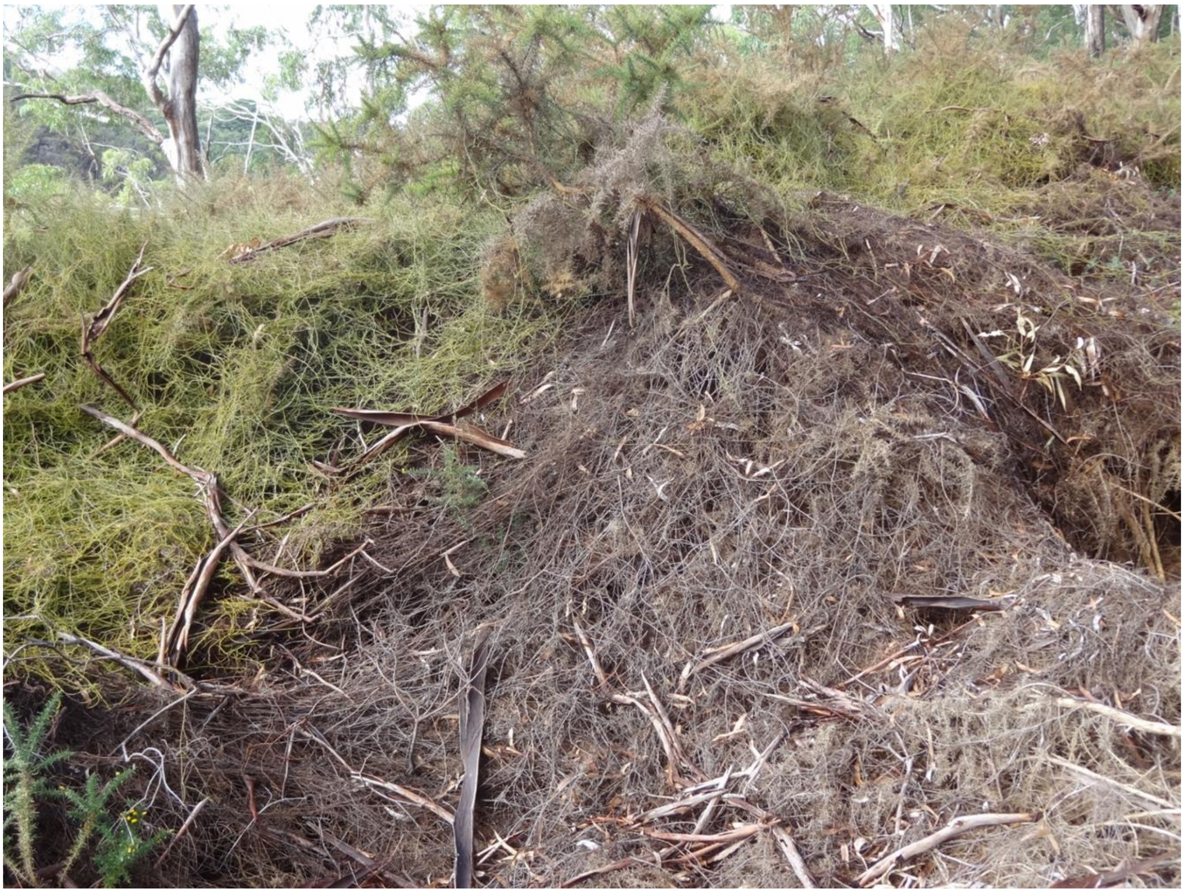

Figure S4. A natural ‘infection front’ of *Cassytha pubescens* moving over a large thicket of *Ulex europaeus* at Crafers (high rainfall area) in the Mt Lofty Ranges of South Australia.

Table S1. *F (italics) and sum of square-values (regular) for independent effects of infection with C. pubescens (I), size of U. europaeus and their interaction (I × S) on total, shoot and root biomass, shoot/root ratio (S/R), nodule biomass (Nod) and Nod g^{-1} host root biomass, predawn and midday quantum yields (F_v/F_m, Φ_{PSII}), maximum electron transport rates (ETR_{max}), shoot midday water potential (Ψ), carbon isotope composition ($\delta^{13}C$), foliar nitrogen (N) and iron (Fe) concentration of U. europaeus. For all parameters df=1, 28 (except Fe: df=1, 12)*

	**Total**	**Shoot**	**Root**	**S/R**	**Nod**	**Nod g^{-1} root**	**F_v/F_m**	**Φ_{PSII}**	**ETR_{max}**	**Ψ**	**$\delta^{13}C$**	**N**	**Fe**
I	*172*	*231*	*66.5*	*6.12*	*37.3*	*7.07*	*15.9*	*5.50*	*10.3*	*0.081*	*14.6*	*0.009*	*18.2*
	101	81.1	21.1	3.51	0.522	1.64	0.031	0.017	0.721	0.006	6.08	0.0009	1.69
S	*22.2*	*23.4*	*15.6*	*1.78*	*7.53*	*2.81*	*1.96*	*4.59*	*2.67*	*4.48*	*12.0*	*1.52*	*5.38*
	13.1	8.23	4.94	1.02	0.105	0.651	0.004	0.014	0.187	0.336	5.00	0.150	0.499
I × S	*4.09*	*3.67*	*3.60*	*0.350*	*5.31*	*0.196*	*0.046*	*0.751*	*0.685*	*1.47*	*1.85*	*0.110*	*22.4*
	2.41	1.29	1.14	0.201	0.074	0.045	0.00008	0.002	0.048	0.111	0.772	0.011	2.07
Error	16.5	9.83	8.86	16.1	0.392	6.49	0.054	0.087	1.96	2.10	11.7	2.76	1.11

Table S2. *F (italics) and sum of square-values (regular) for effect of U. europaeus size (S) on parasite total biomass and total biomass g host^{-1} total biomass, predawn and midday quantum yields (F_v/F_m, Φ_{PSII}), maximum electron transport rates (ETR_{max}), midday stem water potential (Ψ), stem carbon isotope composition ($\delta^{13}C$), nitrogen (N), phosphorous (P) and potassium (K) concentration. df=1, 14 for all parameters (except Ψ, P and K: df=1, 6)*

	**Biomass**	**Biomass g host^{-1}**	F_v/F_m	Φ_{PSII}	**ETR_{max}**	**Ψ**	**$\delta^{13}C$**	**N**	**P**	**K**
S	*61.6*	*0.242*	*0.815*	*1.20*	*0.553*	*0.031*	*1.14*	*14.8*	*14.0*	*62.3*
	3.05	0.088	0.001	0.003	285	0.003	0.400	0.860	0.028	0.769
Error	0.693	5.10	0.020	0.029	7228	0.468	4.91	0.814	0.012	0.074
